# Where are homicide victims disposed? A study of disposed homicide victims in Queensland

**DOI:** 10.1016/j.fsisyn.2023.100451

**Published:** 2023-12-30

**Authors:** Jim Whitehead APM, Richard Franklin, Tracey Mahony

**Affiliations:** James Cook University, Australia

**Keywords:** Search coordination, Search strategy, Homicide victim disposal

## Abstract

Not finding a murder victim poses challenges for homicide investigators in solving crime, including determining where to search for the deceased's body. Existing literature focuses on locating offenders through criminal profiling; however, this is largely based on identification through forensic evidence found at the murder site or where the victim was located. This paper considered the challenge of locating a deceased victim from the perspective of search coordinators assisting homicide investigations. Could reference to previous homicide cases provide patterns and trends that may assist in locating disposed victims quicker, thus aiding in preserving vital physical evidence and providing expedient closure for the community?

**Methods:**

Through generation of a dataset utilising all Queensland Police recorded homicides from 2004 to 2020 inclusive, statistical analysis was conducted using SPSS™ software to identify common trends and characteristics of victim disposal. These identified commonalities were used to develop the Disposed Homicide Victim Matrix (DHVM), and Search Coordinator Principles, as tools to assist search coordinators in future relevant cases.

**Results:**

The study identified four (4) key commonalities observed in the dataset, (1) East is the predominant direction for victim disposal; (2) The offender's vehicle was the most common method of victim transport followed by carrying/dragging; (3) concealment with leaf litter and local debris was the norm, followed by no attempt at concealment; and (4) victims were moved less than 50 m from a road or track after transport.

**Conclusion:**

The DHVM can assist police search for these victims by narrowing down potential search locations. Finding a victim has implications throughout the community, providing evidence that could secure a conviction, allowing a measure of grief closure to the co-victims, and inspiring confidence in police.

## Highlights

• First ‘whole of jurisdiction’ study into disposed homicide victims
• First use of all homicide incidents for a single jurisdiction 2004–2020
• Identified trends and similarities in victim disposal based on relationship between offender and victim
• Development of a Disposed Homicide Victim Matrix to assist searchers
• Matrix has been successful six of eight times

## 1 Introduction

Locating the victim in a homicide was of paramount importance for two reasons. Firstly, for the greater human good, finding the victim allows closure and grieving processes to progress for co-victims [1,2]. Co-victims have identified that the unknown, where the victim has not been found, has a negative impact on grieving and does not allow for closure [3,4]. And secondly, locating the victim aids forensic examination of the victim and crime scene [5]. This may have a flow on effect of identifying further clues for investigators and support the successful conviction of an offender. Conversely, not having located the victim also posed many issues for investigators, not least of which was proving the offence of homicide without a body and identifying a suspect for the crime [2].

There was no formal definition of a no-body homicide but Dibiase [2] identified it as a homicide where the victim has been hidden or destroyed. An exploration of Australian no-body homicides by Ferguson et al [6,7] noted the difficulty for the prosecution to prove the offence elements and obtain a successful conviction when the victim had not been found. While much had been written on victim disposal after a homicide had occurred [[8], [9], [10], [11], [12], [13], [14], [15]] those studies had been conducted on small homicide subsets, such as sexual serial killers, familial killings or rural murders, and did not focus on cases of victim disposal (no-body homicides).

In addition, the challenge of finding victims in a timely manner were encountered as many murder investigations often commence with the victim initially being reported as a missing person. When the victim is a missing adult, there can be a time lapse of hours or days before a homicide is suspected or confirmed by police. This meant searching in homicide incidents often did not commence for several days as other inquiries were initially required by undertaken by investigators. In this lapsed time the opportunity to collect vital forensic evidence can be damaged or lost.

Literature on no-body homicides can be broken down into five themes: sexual serial killers; serial killers; once-only killers; offender forensic awareness and disposal patterns. One Australian based article was at the focus of this research, Ferguson et al [7] explored no-body homicide convictions and found that homicide investigation was stymied without the main source of evidence, the victim. While a conviction for a no-body homicide was not impossible the article did emphasise that there was a higher chance of the incident not being brought to a successful conclusion because the victim had not been located. This conclusion was also reached by Dibiase [2] who similarly explored no-body homicide convictions in the USA. Neither article attempted to suggest methods of victim location.

Four articles aimed at disposal patterns and linkages to offender profiling within sexual serial killings in the USA and Canada were located ([5,8,11]; Snook et al., 2005). These were based on the finding of a victim and subsequent analysis of the crime scene to identify possible offenders. The articles did not reference distances travelled, methods of disposal and concealment except in the broadest terms. In all instances the victim was located, but the articles did provide evidence of commonality within victim disposal, but no suggestion as to whether the method of homicide had a bearing or influence on victim disposal.

Sea and Beauregard [5] explored 57 South Korean homicide incidents, resulting in a comparison of disposal sites, distances and relationships with respect to victim disposal. The strength of this paper was the conclusion that there were similarities in disposal choices and distances travel to the disposal site. The major limitation was the small sample size compared to the overall homicide numbers in South Korea. The authors also identified that disposal methods appear to underpin a level of forensic awareness by the offender.

The literature review identified no studies focusing on homicide victim disposal from a single policing jurisdiction. There was also no known literature that identified the percentage of homicide victims that had been disposed compared to the total number of homicides for any Australian policing jurisdiction. Therefore it is not known if Queensland, with 147 disposed victims, 19.6 % of the total homicides for the study period, represented the wider Australian population. It has to be noted that some cases may still be recorded as missing persons rather than homicides due to a body not being found.

As search and rescue coordinators were often called to help find homicide victims, there was a need for guidelines. A gap in knowledge of victim disposal, around any trends or commonalities in the moving of a victim from where they were killed to a secondary location has been identified [16].

This study examined all homicide victim disposal incidents in a single policing jurisdiction to describe common victim disposal actions. The information from the victim disposal analysis was the basis for the Disposed Homicide Victim Matrix (DHVM), developed to help find victims more often and faster. This would have the flow on effect of reducing no-body homicides, preserving forensic evidence, increasing opportunity for successful prosecution of offenders and hastening subsequent processes closure for families and the wider community.

## 2 Methodology

Homicide records sourced for this study were obtained with the permission of the [17] (QPS) Research Committee and under James Cook University Ethics Approval H7197. Homicide records from the QPS were stored in a consolidated electronic repository called the Queensland Police Records and Information Management Exchange (QPRIME). The QPS adopted QPRIME in 2004, replacing multiple older intelligence systems, and was the sole repository of all crime data for that jurisdiction. QPRIME adoption dates determined the start of data collection and the final year, 2020, was determined by those homicides that had been solved and/or finalised through the court system. Access to the data contained within QPRIME was undertaken for the sole purpose of this study, and was completed during the period 2018 to 2022. Incidents were accessed using the keyword offence term of ‘homicide’ and ‘manslaughter’.

While there was a significant amount of data contained in QPRIME, the system functionality limits search ability to a few fields, such as incident type classification, offence date, offender and/or victim, location address and similar. As identified by Ferguson et al [6], QPRIME access was not afforded to other researchers in this field and thus the dataset provided a unique perspective on homicides in this jurisdiction.

Within each QPRIME homicide entry was all the information and intelligence collected throughout the investigation, including case summaries, witness statements, forensic analysis, search efforts and victim/offender biometrics. It was from these case files that the data for this study was collected.

Coding of the homicide data was required and the codes used can be found in Table 1. The coding categorised like data together when there were a range of differences within a small data set. An example of this occurred in strangulation as a murder method, which had belts, rope, wire, ties and stockings used as the actual tool involved. This type of coding was required as many of the data sub sets had small numbers involved.

**Table 1:** Homicide data coding.

Demographic	Code	
Offender/victim sex	F = Female M = Male	U = Unknown
How was the victim located?	M = Medical response N = Never located O = Organised search OF = Offender identified location	P = Police response R = Randomly found (Bushwalker, farmer etc) W = Witness
What was the method of homicide?	B = Blade weapon (Knife, machete, axe etc) BL = Blunt trauma weapon (Hammer, metal pipe etc) C = Chemical (Poison, acid, medication, intravenous substance etc) D = Drowning E = Electrical EX = Explosives F = Fail to provide necessities of life (Starvation, dehydration, deprivation of liberty etc)	FA = Fall (Pushed off cliff, building, bridge, aircraft etc) FI = Firearm (Hand gun, rifle, shot gun etc) FO = Firearm other (Hand-made gun, single use gun) I = Immolation M = Motor vehicle P = Physical assault (Beating, one punch etc) ST = Strangulation (Rope, belt, wire etc) SU = Suffocation (Pillow, plastic bag, gag etc) U = Unknown
How was the victim disposed?	C = Concealed Insitu (Not moved and covered by objects at incident location, carpet, plastic sheet, furniture) CG = Concealed on ground (Moved from scene and covered in leaf litter, soil, branches, wood, tin, debris etc) CO = Concealed other (Moved from scene and placed in wheelie bin, industrial bin etc) CR = Cremation D = Dismemberment	I = Interment (Burial at depth, purpose dug grave) N = Never located NI=No concealment insitu (Not moved and left uncovered at incident location) NM = No concealment (Moved from scene and left on open ground, paddock, roadside etc) S = Shallow grave WS = Waterside (Beach, river/creek bank, dam wall etc) WW = Waterways (In ocean/sea, river, creek, pond, dam, drain etc)
Method of victim transport?	N = Not moved C = Carried/dragged M = Motor vehicle (Off/victim)	O = Other motor vehicle U = Unknown V = Vessel

The dataset was also categorised based on the relationship between the victim and offender. In this study, the offender and victim relationship in those instances where the victim was disposed have been classified into three (3) categories, (1) Acquaintances, (2) Familial relationships and (3) No known relationship. The description of the relationship types within the three broad categories are shown in Table 2. The No known relationship category was not as a result of missing data but that there could be no relationship established between the victim and offender, a totally random homicide.

**Table 2:** Relationship description.

Category	Description	N (%)
Acquaintance	Acquaintance, Co-worker, Employee/Employer, Employer/Employee, Friend, Neighbour,	71 (48.3 %)
Familial	Boyfriend/Girlfriend, Daughter/Father, Daughter/Mother, De-facto, Engaged, Father/Daughter, Father/Son, Mother/Daughter, Mother/Son, Other relative, Same sex relationship, Sibling, Son/Father, Son/Mother, Spousal (Estranged), Spousal (Married)	54 (36.7 %)
No known relationship	No known relationship	22 (15 %)
Total cases		147

It was determined that the start point for any analysis of transport and distances was the site of the murder. While this was not always an exact location such as a house address, there was sufficient information within the case files to identify the scene if in a rural location. No murder site was used more than once during the period of this study, but what was identified as important was distance and direction that a victim was disposed from that site. It was in the direction and distance that any patterns or trends would be found in victim disposal. The distance and directional measurements between the homicide site and the victim disposal site were plotted on Google Earth Pro™ and entered into the Excel™ spreadsheet. The direction of victim disposal was reduced to the eight major compass directions (north, north-east, east, south-east, south, south-west, west, north-west) rather than degrees.

Only empirical data was collected from QPRIME, such as dates, locations and relationships. It was considered that the motivation for victim disposal would be relevant to this study but as there was no need of the prosecution to prove motivation or rationale to secure a conviction this was not always included within the case files. Further research may identify the nexus between motivation and disposal.

A cleanse of the QPRIME data was conducted applying inclusion and exclusion criteria. The four inclusion criteria for the dataset was (1) that the offence was a homicide or manslaughter initially to ensure totality of the data (2) that the victim had to have been disposed (i.e. moved from the scene); in some means; (3) the incident occurred between 2004 and 2020; and (4) the incident occurred in Queensland as this was a single jurisdictional study. The three exclusion criteria for the dataset were (1) that no mass killings were included; (2) no serial killings were included; and (3) homicides where the victim was not moved from the crime murder site. Serial and mass killings were identified as often having a clustering effect, that the victims were disposed in the same or similar locations depending on the offender's [8]. The QPRIME incident number for each homicide was used in the data cleaning process to ensure no duplication of incidents. A total of 147 homicide incidents met the criteria to form the dataset for this study.

## 3 Findings

The dataset contained 147 instances where the victim's body was moved after a homicide, of which 130 had been located, 88.4 %, the remaining victims had not been found at the time of this study.

### 3.1 Finding victims

Of those victims located, the majority were found during a police response, such as welfare checks or through organised searching of a location, 60.5 %. The second method of locating a victim was through a ‘random find’, for example, location by bushwalkers or farmers working in their fields at 15 %. The offender's themselves had provided information as to the disposal site in 6.8 % of cases, and witness information accounted for 6.1 % of cases.

### 3.2 Victim disposal directions

**Fig. 1:**
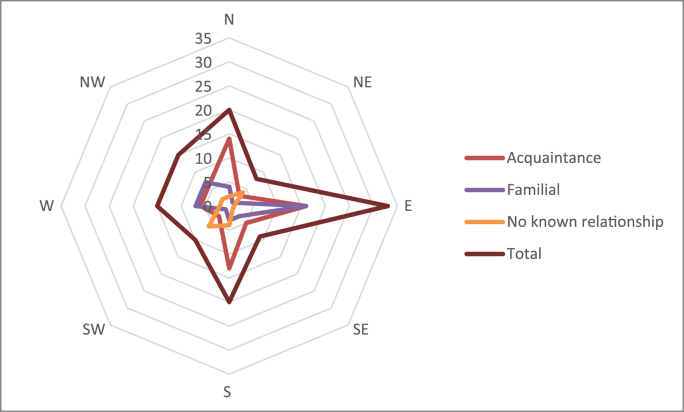
Victim disposal directions by relationship.

Fig. 1 depicts the disposal directions for each homicide case in the dataset. East had the largest number of victims disposed 25.4 %, with north and south 15.4 %, west and north-west 11.5 %, south-west 7.7 %, south-east 6.9 % and north-east 6.2 %. There were a further seventeen victims that have never been located, making it impossible to determine their direction of disposal. The victim disposal directions for the three relationship categories can also be found in Fig. 1.

While east was predominant disposal direction in familial and acquaintance relationships, south-west being the most frequent direction of disposal at 27.3 % in those cases of no known relationship.

A theory by Bond [18] was that modern humans had evolved from being home centric, being able to walk home from any location such as First Nation people on walkabout, to being north centric based on the compass. This meant that in most cases it was east or north, based on our ability to identify either through sunrise direction or compass bearing. This process may impact on the direction chosen by an offender when disposing of their victim.

### 3.3 Victim relationships and disposal distances

Victim disposal transportation distances from the initial murder site ranged from 50 m up to 730 km. The median and average with a 95 % CI at which the most number of victims were disposed of was up to 100 m. This was followed by 110 m–500 m, 15.4 %, 1–5 km 12.3 %, 11–15 km 10.7 % and 6–10 km 8.5 %. For disposal distances of 15 km and under there were no instances where there was a sole victim. Transported distances above 15 km at which two victims were disposed was 6.9 %, with three and four victims at 3.1 %. There were seven distances over 100 km, each with a single victim.

The most frequent distance a victim was moved from a road/track after transport to the disposal site was 10 m (42.2 % overall, 43.7 % for acquaintances, 35.2 % for familial and 54.5 % for no known relationship), which is basically moving a victim from a vehicle to the side of the roadway. The next two frequent distances were 20 m 22.4 % and 30 m 11.6 %. The longer distances, over 30 m, were used rarely 5.5 %. The average distance a moved victim was located from a road or track is 17.37 m 95 % CI.

The acquaintance relationship category had the highest number of victims removed from the incident site 48.3 %, and also the largest range of the movement options at four, although there were a small number where the method was unknown, Table 3. Almost half of acquaintances, 49.2 %, used their own motor vehicle to dispose of their victims compared to 42.6 % of familial and 41 % of no known relationship. Carrying or dragging the victim was higher within the no known relationship category, followed by familial and acquaintance, 54.5 %, 37 % and 35.2 % respectively. Victim movement in the no known relationship category was limited to carrying/dragging and offender or other persons vehicle, while the familial category had the most instances where the movement method was unknown, 20.4 %.

**Table 3:** Method of victim transport by relationship.

Method of transport	Acquaintance	Familial	No known relationship
Carried or dragged	35.2 %	37.0 %	54.5 %
Motor vehicle (Offender's, victim's)	49.3 %	42.6 %	41 %
Motor vehicle (Other persons, not co-offender's)	7 %	0 %	4.5 %
Unknown	7 %	20.4 %	0 %
Vessel	1.5 %	0 %	0 %
Total	100 %	100 %	100 %

### 3.4 Demographics of homicide offenders

Males represent the greatest proportion of offenders who moved their victim at 86.4 %, and also the largest percentage of victims, 55.1 %. Females account for only 7.4 % of offenders and 44.9 % of the victims. Unknown offenders represent 6.1 % of the total number. Males were the most frequent movers of victim's bodies, moving 65 male and 62 female victims. Females only moved three female and eight male victims. In nine cases it was unknown who moved the victims.

### 3.5 Concealment in disposal

The most frequent concealment method was simply covering the victim with whatever was proximate to the disposal location, such as leaf litter, branches and debris with 51 victims, 34.7 %, and was most frequent within acquaintance and familial relationships. The next grouping, 14.3 %, was to leave the victim with no attempt at concealment. Most of these victims appeared to have been removed from a vehicle and left on the side of a road or track.

Where there was no known relationship, concealment was almost evenly split between the above two methods. In seven instances, 4.7 %, the victims were disposed in a wheelie bin, industrial bin, and on one occasion each a large tool box and a 44 gallon drum. Shallow graves, where the victim was put into a man-made or natural hollow in the ground, accounted for 12.2 % of victims, with a further 4.1 % being interred at a significant depth. Cremation or attempted cremation occurred on 6 occasions, 3 in the acquaintance category, 2 in the familial category and the last victim was in the no known relationship category. The single dismemberment was from an acquaintance relationship, Table 4.

**Table 4:** Victim concealment method by relationship.

Concealment method	Acquaintance	Familial	No known relationship
Concealed on ground (Moved from scene and covered in leaf litter, soil, branches, wood, tin, debris etc)	36.6 %	35.2 %	27.3 %
Concealed other (Moved from scene and placed in wheelie bin, industrial bin etc)	9.9 %	5.6 %	9.1 %
Interment (Burial at depth, purpose dug grave)	4.2 %	5.6 %	0 %
No concealment (Moved from scene and left on open ground, paddock, roadside etc)	12.7 %	9.3 %	31.8 %
Shallow grave (Buried in man-made/natural shallow excavation)	14.1 %	11.1 %	9.1 %
Waterways (In ocean/sea, river, creek, pond, dam, drain etc)	9.9 %	7.3 %	18.2 %
Other	5.6 %	3.7 %	4.5 %
Never located	7 %	22.2 %	0 %
Total	100 %	100 %	100 %

### 3.6 Homicide type

Bladed weapons, blunt trauma weapons and physical assault accounted for half, 52.3 %, of all homicides. Eighteen offenders chose strangulation, while firearms were used on 8.1 % occasions. A cause of death was unable to be determined for 19.7 % of victims, as this also included those that had never been located. Further analysis identified that bladed and blunt trauma weapons were favoured by females, 28.8 %, followed by physical assault, 19.7 %, and suffocation, 18.2 %. The physical assault was against babies and young children, with suffocation equal between children and spouses. Firearm usage by males, females and unknown is nine, two and one respectively in instances where the victim was moved. Men more commonly used blunt force trauma weapons, 19.6 %, bladed weapons, 18.5 %, physical assault, 17.3 % and strangulation, 12.3 %.

The murder occurred in the offender's residence on 34.7 % occasions and the victim's residence a further 31.3 % occasions. The remaining 50 incidents occurred at locations such as parks, wilderness, and work places or are simply unknown.

## 4 Discussion

This was the first known study into the disposal of homicide victims within Australia, and although limited to Queensland accounts for approximately 24 % of Australian homicide incidents in the period of this study, 2004–2020 [19]. Based on the information gathered, a Disposed Homicide Victim Matrix (DHVM), Table 5, had been developed. This matrix provided information on which a search strategy could be formed, resulting in the development of statistically viable search areas where the victim was likely to be located.

**Table 5:** Disposed homicide victim matrix (DHVM).

	Acquaintance	Familial	No known relationship	Average (All homicides)
**Distance from homicide site km**				
Mean (All murders)	22.76	30.90	4.67	19.44
Std. Deviation	62.47	114.72	6.27	61.15
95 %	147.7	260.34	17.21	141.8
Mean (<100 km)	8.13	5.82	4.67	6.21
Std. Deviation	16.33	11.62	6.27	11.41
95 %	40.79	29.06	17.16	29.0
Mean (<51 km)	3.7	4.48	4.67	4.28
Std. Deviation	6.28	8.36	6.27	6.97
95 %	16.26	21.2	17.16	18.2
**Method of transport (Most common)**				
Motor vehicle	49 %	49 %	41 %	46 %
Dragged/carried	35 %	43 %	55 %	44 %
**Method of concealment**				
On ground, covered with leaf litter, branches and/or debris	37 %	35 %	27 %	33 %
Shallow grave	14 %	11 %	9 %	11.3 %
Concealed other	10 %	6 %	9 %	8.3 %
No concealment	13 %	9 %	32 %	18 %
Never located	14 %	22 %	0 %	12 %
**Average distance moved from transport**				
Distance (m)	17 m	17 m	17 m	17 m
**Direction taken to dispose of victim**				
Most common three directions	East	East	South	East
	North	North	South-west	North
	South	West	North-west	South

The DHVM could be used by a search coordinator to define an initial search area based on the relationship between the offender and victim. In the case of an acquaintance relationship a circle of 16.26 km radius could be drawn around the murder site, representing 95 % of all disposed victims in that category, and 92 % of all disposed victims overall. Within this circle roads going the three prominent directions of east, north and south could be identified as a motor vehicle was the most common form of victim disposal transport. Areas that were not often frequented by other people could be subsequently identified adjacent to these roads, including conservation parks, forests and the like. The data suggested that victims were moved an average of 17 m from a road/track but no more than 60 m, negating the necessity to initially search large forest or farmland areas. Victim concealment, in the form of covering with ground litter, formed the basis of a searcher briefing. While not providing an exact victim location, the DHVM did provide viable search areas that could be refined by intelligence gathering techniques.

### 4.1 Method of homicide

There were a number of different homicide methods identified within the dataset, with most being with weapons of opportunity such as bladed and blunt force trauma items close at hand. This corroborates that most homicides were being committed in the heat of the moment and were unplanned [1]. Access to firearms was strictly limited within Queensland, and the firearm legislation restrictions were reflected in the relatively small number of homicides using this method, 8.2 %. At odds with Lee et al [11], strangulation was not the most prolific murder method of those victims that were disposed. Within the individual relationship categories, blade weapons had the highest usage rate among acquaintances which may possibly equate to the increased carrying of bladed weapons within the community [20]. While it was possible that some of the murders within the dataset were premeditated Brookman [1] and DiBiase [2] suggest that there was a very fine line between threatening behaviour and actual killing, and that in the heat of the moment this could be crossed without any conscious knowledge that it had been. The information contained within the dataset did not provide any evidence of significant planning in the murder and disposal of any victim.

### 4.2 Victim concealment

Human bodies are difficult to dispose, representing a literal dead weight if deceased or unconscious that is awkward to move alone. A body is difficult to dissect or dismember without some medical knowledge and it takes an industrial furnace at 800–1000 °C to cremate properly [21]. Those difficulties explain in part why homicide victims were often not moved from where they were killed. As identified by Brookman [1], limited planning accompanies a murder, and this was borne out in the dataset, with 57 % of disposed victims either being left on the ground where they were transported to or placed into waste receptacles. For those left on the ground concealment was limited to covering with locally available material such as vegetation, sticks or logs, sheet metal or rubbish. Disposal in this manner allowed access to the victim by predatory animals, often resulting in the scattering of remains over time. It further aided searchers, in that the victims could be better identified as they differed in shape, form and colour from the background [16]. Time and location could affect victim concealment to the extent of leaving the victim on the side of a remote or rural road, a task that could be completed in minutes. While bushfires can exceed 1600 °C in extreme conditions a small fire created around a disposed victim would generate insufficient heat to cremate a human body [21], and as a result all disposed victims where cremation was attempted were located relatively intact and recognisably human as opposed to an ash mound.

There were two aspects of victim concealment identified in the literature that impact on locating disposed victims; forensic awareness of the offender and concealment of the body by the offender. Forensic awareness had been described as an offender modifying their actions when committing a crime to limit the transfer of evidence and reduce the chances of being caught [22]. Forensic awareness had been suggested as contributing to victim disposal, both from an association avoidance technique and a means of forestalling any investigation perspective [[22], [23], [24], [25]]. Within this study there was no evidence that offenders exhibited any forensic awareness apart from moving the victim from where they were killed. Other forensic awareness actions could have included wearing disposable clothing, not using their own identifiable property to wrap or conceal the victim and to limit DNA transfer through using gloves, masks and hair nets, none of which were evident in the police reports. The majority of homicide and transportation methods used offered the potential for forensic testing to be successful, such as leaving evidence at the initial site, inside a vehicle, at the disposal site and on those involved [23,26].

### 4.3 Victim location

Six out of 10 victims were located through a police response, such as a welfare check, missing person inquiry or organised search. This aligned with Ferguson et al [7] who identified that no-body homicides often started as missing person reports and initial investigations are often left with Search Coordinators or general duties police for follow-up. Physical searching had been the major contributing factor to locating disposed victims, often conducted sometime after the actual homicide and at the instigation of the investigator. Searching had been based around intelligence gathered during the investigation and from witnesses. It was a very laborious process as precise locations were rarely identified and any remains may be impacted by environmental conditions, predatory animals and other interference [16]. Under the ‘no body, no parole’ laws some offenders have led police to the disposal site (“[27]," 2006) many years after the murder.

### 4.4 Victim disposal directions

A map of Queensland identifies that the majority of the formed roadways run basically north-south or east west. The major highway system follows the coast the entire length of the state with other highways branching off westwards. The major connecting roads then link these highways in an approximate north-south direction. Due to the infrequency of victims being transported great distances, such as over 50 km, it would be reasonable to utilise the average distances in the initial stages of a search for a missing homicide victim, and refine the area as further intelligence and information became available. With a motor vehicle being identified as the most common method of victim transport this allowed strategies to be immediately implemented to narrow down the travel options through the use of Automatic Number Plate Recognition (ANPR) and Traffic Monitoring Cameras (TMC).

Field and Beauregard [28] had identified that most homicide offenders tended to adhere to known locations or routes. Given that the majority of Queenslanders reside on or near to the east coast it seemed logical that an offender would head in that direction to dispose of a victim. This would also apply to going north or south. There is no obvious causal factor restricting offenders going north-east or south-east as identified, except perhaps the limited number of roadways that go in those directions and/or the lack of knowledge of them. Notwithstanding this, it was possible for a determined offender to travel in any direction of their choosing although this had not been borne out by the research.

The Great Dividing Range, which generally parallels the eastern coast of Queensland, had been identified as a potential barrier to victim transport directions. There were 5.5 % of the total victims killed on the western side of the Great Dividing Range, of which only 12.2 % were disposed. This represents less than the Queensland average, more than likely due to the sparseness of the population when compared to the more populated coastal areas. The topography west of the Great Dividing Range was generally flat, with limited sealed roadways but numerous secondary thoroughfares, potentially providing disposal routes in all directions. The DHVM suggests that an appropriate search radius around the murder site be identified and searching concentrated on the roadways going east and north initially, out to 60 m either side.

### 4.5 Victim disposal distances

Sea and Beauregard [5] studied 54 murders in South Korea, identifying distances from the first contact site, the offenders residential address and murder site to the disposal site, with 87 % of offenders disposing of their victims within 30 km of the murder site. A previous study by Häkkänen et al [10] on sexual serial killers found that they disposed of their victims up to 50 km from the murder site but also that they could be disposed beyond 200 m from an official roadway. The Queensland located disposed victims were taken distances ranging from 100 m to 730 km from the murder site. Those distances were measured in a straight line as in most cases the exact route of travel was not known or recorded. Interestingly, of the seven greatest distances four were acquaintances and three were estranged spousal relationships, representing total opposites of the relationship spectrum, differing with no known relationship, which have much shorter disposal distances. There may have been some long term animosity or hatred between the parties, prompting the offender to inflict further distress on the victim's families by moving them so far they would be unlikely to be found [29]. This may also correlate with the rational choice theory [30], that the offender rationally chooses the distance and location of their victim disposal to maximise self-interest, such as not being caught.

The most frequent distances a victim was carried or dragged from the crime scene were up to 100 m, this is about as far as an average person could drag or carry a deceased person according to McCluskey [31]. No victim was dragged or carried more than a kilometre from the murder site, and no motor vehicles were used for any distances less than 50 m. Within all three relationship categories, 84 % of disposed victims were located within 15 km of where they were killed. Depending on the location it could be less than 1 h of travel by motor vehicle.

Male offenders aged between 26 and 45 represent over half of all victim disposal, with an equal statistical spread throughout the four age groups involved. Without supporting evidence, it would be reasonable to attribute this to a males’ most physically strongest years, giving them the most strength to move victims during the act of disposal. A similar situation exists among females, the highest incidence over the same age group, [32]. There was a distinct age bracket of 21–55 years, representing 75 % of all moved victims. This also correlated with the age bracket for the highest number of homicides victims. Male and female victims showed similar patterns of disposal in all age groupings and there was no identified statistical significance between any particular groups.

### 4.6 Distances moved from transport

Those victims dragged from the murder scene were either to a neighbouring property or away from the murder location on the same property, such as a garden or back yard and were almost always, 91 %, along a fence line or pathway. In all instances where a body moved from a vehicle to a disposal location it had been in a downward direction. There wre no recorded instances of a body having been taken up hill, and only one found outside the research [33].

The furthest distance a victim had been located from the nearest road or track was 60 m, this is inconsistent with Häkkänen et al [10] who suggested distances up to 500 m were possible from official roads. The differences may be an interpretation of what is an official road, as from the dataset it was the nearest road/track, not necessarily a formed official road. Keppel et al [34] also suggested that victims would be found within 15 m of a road or trail and the most common distance were between 10 m and 30 m, which was similar to the dataset findings. In the disposal areas there had generally been bushland edging the roadway, offering a measure of concealment and delaying the finding of the body. Estimating distances in a bushland setting was difficult, perhaps contributing to offenders believing they had gone considerably further than in reality [35]. The research indicates that victims were never disposed in the centre of large acreages or areas, always around the peripheries.

### 4.7 Concealment

Basic concealment of the victim on the ground and covering them with nearby materials was the predominant method of disposal. Although easier, making no concealment after disposal accounted for less than one seventh of all disposed victims, 14.2 %. Knowing the type of concealment would enable suitable search tactics, such as utilising both visual and electronic searching of potential areas of disposal, to be employed. The data indicated that most victims were left relatively close to roads and fences, and it appears that offenders relied heavily on choosing remote or less populated locations for victim disposal. The direction a victim was taken provided an indication of where to search, although it must be remembered that those are straight line directions between a murder site and a disposal site. By necessity, an offender may not be able to travel in a direct line, having to follow roads and tracks.

### 4.8 Challenges for investigators

There are a number of challenges facing investigators when looking for victims in no-body homicides, not least of which is where to search. If information was not forthcoming from the offender or the offender was unknown it was difficult to progress further. Both DiBaise [2] and Brookman [1] identified that the victim was paramount from both an evidentiary and closure point of view. Literature is scant on the actual search process but did provide tactics for investigators to gather intelligence that may contribute to developing a search area [1,2]. The data collected through disposed victim homicides where the victim had been subsequently located was the basis for the Disposed Homicide Victim Matrix. The challenge for investigators was to gather sufficient data about the incident to make using the matrix a viable option.

### 4.9 Guidance for search coordinators

The study had generated a DHVM, Table 5, which showed what actions offenders have taken in previous homicide cases involving victim disposal. Generally, in behavioural models, past behaviour was a good indicator of future behaviour [36]. It was therefore proposed that the DHVM could be used as a starting point for search coordinators when considering the most likely actions taken by an offender. From the findings of this study, a list of general principles had been formulated to provide guidance for search coordinators when considering the location of a disposed victim. The general search conclusions that could be drawn from Queensland disposed homicide victims are contained in Table 6.

**Table 6:** Search strategies principles for disposed victim homicides.

**1**	The method of homicide is material in understanding the effects of decomposition on the victim and the type of searching required.
**2**	For those victims moved the most common method of disposal is dumping them on the ground with no effort in concealment, followed by concealment with items to hand such as logs, branches and leaves. Shallow graves in the form of small man-made or natural depressions and cremation are next in order of occurrence.
**3**	Searching by police, using current methods, is the most successful means of locating moved victims.
**4**	The victim has been disposed up to 12 km from the place of homicide in 75 % of all incidents,
**5**	The furthest a victim has been left from a road or track has been 60 m.
**6**	East is the most prominent direction for disposal overall, and for six of the fourteen relationship categories involved.
**7**	Weight disparity does not appear to have an influencing effect on victim disposal.

A previous study on search strategies [37] identified that those used to locate lost and missing people could readily be adapted to the search for disposed homicide victims. The actual search patterns and type of searching undertaken is dependent on the terrain, topography, vegetation and time elapsed since the victim disposal, and can be found in the National Search and Rescue Manual [16].

### 4.10 Limitations

While all reported Queensland homicide incidents were collected as part of the initial dataset, Mouzos (2002) suggested that this may not represent all cases. Murders not recorded as such could be missing people without evidence of murder, unrecognised murders attributed to a medical ailment, murders in aged care facilities and incidents involving gang type homicides. It could be argued, that at 19 % of all Australian homicides, any findings and conclusions may not be representative across the whole country. As identified by Hendra et al [38] a comparison of the respondent sample and full sample would produce a zero difference as the full sample was initially conducted, representing a nil impact bias.

The calculated distances used in the study were Euclidean, that is, a straight line between the murder site and the victim location site. The Manhattan distances, used in geographic analysis, is the sum of all the real straight line distances between both sites [39]. In reality there can be more than one Manhattan distance. The Euclidean distance was used as the route was not always recorded in the homicide case record. Further studies could focus on the Manhattan distances to enhance the matrix.

This scope of this study was delimited to Queensland. This research provided the basis for a larger study to identify if Queensland was representative of Australian homicide victim disposal or does the large size and multitude of land forms, cultural range, population densities and socio-economic situation make this state unique. While the number of homicide incidents was low, this study did utilise the entire available dataset and could only be enhanced by a full national study, which would be an opportunity for further research. This research has management implications for searching and could be used to determine areas that may maximise the locating of undiscovered victims.

### 4.11 Implications for theory

The results of the research support the contention that homicide offenders tend to dispose of their victims in line with the matrix, that is, within a range of parameters associated with their relationship to the victim.

The theory that the disposal of homicide victims has commonalities in methods, locations, distances and concealment has been borne out with the research. It has been shown that the demographics of victim movement and subsequent concealment can be grouped into relatively broad clusters that have few outliers. The results support the use of the Disposed Homicide Victim Matrix in planning for searches and the prediction of suitable search areas. The matrix demonstrated that while very broad, the existing literature around victim disposal is grounded. It also demonstrated that a state-wide examination of homicide victim movement data was possible as a means of confirming smaller data sets such as relationship types that ultimately impact on the victim disposal.

### 4.12 Implications for practice

The Disposed Homicide Victim Matrix provided a statistical reference in the search for unlocated victims. Being trend and commonality based it represented what the majority of killers had done with their victims previously, and while there were many similarities there were also individual traits that could not be accounted for. The matrix provided the biggest single leap forward in search planning for disposed homicide victims aside from the offender actually identifying the disposal site. At the time of writing, the matrix had been used on eight occasions, with six homicide victims being located.

The seven homicide victims, from six incidents, were located in four Australian states, Australian Capital Territory, Victoria, Tasmania and Queensland. Five of these homicides are still before the Courts and are sub-judice and therefore only basic details can be provided. The remaining case was the search for Victim 1 killed near Gatton.

#### 4.12.1 Victim 1

The search for Victim 1, a 15 year old female, was commenced several days after her disappearance. Using the last known location, information about the offender and the topography of the area a number of potential search areas were identified within a 17 km radius of Gatton, Queensland. The victim was located approximately 20 m off a lonely country road, covered with grass from recent hay making, thirteen days after her disappearance. She was south of the murder location. It has to be stressed that this search was based on a behavioural model theorised at the commencement of this project.

#### 4.12.2 Victim 2

Victim 2 was murdered in his home in suburban Canberra. A suspect had been identified through further investigations but had refused to cooperate with police. The Australian Federal Police requested assistance with possible search locations, and subsequently a number of parameters were provided that could be potential victim disposal sites. Acting on this information, police were initially unable to locate the victim after searching areas that fitted within the parameters. Based on what locations had been searched, a refinement was made and police were redirected to a forest area near the Mount Majura Nature Reserve were the body of the victim was located. The straight line distance was 9 km in a south by east direction with the victim being 10 m from the nearest track. There was no attempt at concealment. A period of fifteen days had elapsed since the murder. The initial location was incorrect based on information supplied, but was able to be corrected with a change to the relationship category.

#### 4.12.3 Victims 3

Husband and wife victims 3a and 3 b disappeared from a camping ground in north eastern Victoria. Investigations by the Victoria Police Missing Persons Unit uncovered evidence of ongoing arguments with a fellow camper. It was believed that the suspect had disposed of the victims in bushland and had returned at a later time to burn and scatter what human remains remained. A number of search areas were developed using the matrix and forwarded to Victoria Police. Subsequent searching by police and volunteers located the remains of both victims within one of the areas targeted, approximately 7 km east from the murder site and a short distance off a fire trail. Concealment was initially by covering with vegetation but after revisiting the site body parts and cremated remains were scattered in the vicinity.

#### 4.12.4 Victim 4

Female victim 4 was last seen walking across a bridge over a river in northern Tasmania. Initially reported as a missing person, police inquiries soon identified that she may have been the victim of homicide. Six weeks after the disappearance police sought assistance in the search. A number of potential search areas were identified around the disappearance site. Two weeks later police located the victim concealed under two logs, approximately 7 m off a small track within a Forest Reserve to the east. The victim was located 100 m short of the average distance provided, within the 15 m from the road/track and concealed as predicted. The direction of disposal was slightly north of east.

#### 4.12.5 Victim 5

Female victim 5 disappeared from her north Queensland home under suspicious circumstances, and with her physical disabilities it was totally out of character. She was initially reported as a missing person, with a criminal investigation running parallel. Information was sought on possible locations if she had been murdered and a number of locations were identified for searching. Fifteen days after her disappearance, she was located in dense bushland within an identified search area, 4 km south-east of the murder site. There was no attempt at concealment, perhaps because of the remote location.

#### 4.12.6 Victim 6

Victim 6 disappeared from her home, which she shared with her killer. Investigations into the disappearance were initially undertaken by the Missing Persons Unit until it was suspected that foul play may have occurred. Assistance was sought with respect to potential search areas which were identified within the Glass House Mountains area. Prior to a formal search being conducted the victim's remains were located in a random find by four-wheel drivers in a forest, 21 km north-east of the murder site, less than 10 m from a four-wheel drive track. There was some charring of the body but it was otherwise unconcealed.

There were two other instances where the matrix was unsuccessful in locating a victim, but this may have been a combination poor search coordination or the disposal being outside the identified normal range of offender actions.

## 5 Conclusion

While the challenge of locating disposed homicide victims remains, this study had identified that there were commonalities with the disposal of victims. The relationship between the offender and victim was vital as it directly impacted the direction and distance of victim disposal. The study identified that east is the predominant victim disposal direction and that the average disposal distance for all homicides where the victim was disposed was 19 km. The average distance for the 89.2 % of victims who were disposed at less than 50 km was 4.28 km from the murder site. For those victims moved from a vehicle at the disposal site, 17 m was the average distance they had been taken from a road/track with no victim being found beyond 61 m.

The offender's motor vehicle was the most common method of victim transport, followed by carrying or dragging. Covering the victim on the ground with items close at hand was the most common method of concealment followed by no attempt at concealment at all. Those commonalities had been translated into the Disposed Homicide Victim Matrix which could assist police search coordinators determine statistically viable search areas based on what had happened previously. The Matrix also identified that all located victims had been found relatively close to roads or tracks, negating the necessity to search beyond 100 m from these in the first instance.

## Funding

No funding was provided for this research.

## CRediT authorship contribution statement

**Jim Whitehead APM:** Writing – original draft, Writing – review & editing. **Prof Richard Franklin:** Supervision. **Dr Tracey Mahony:** Supervision.

## Declaration of competing interest

The authors declare that they have no known competing financial interests or personal relationships that could have appeared to influence the work reported in this paper.
